# A qualitative exploration of the health system responses to the screening and management of comorbid mental illness and chronic physical illness in Jamaica

**DOI:** 10.1371/journal.pone.0290975

**Published:** 2023-12-14

**Authors:** Patrice Whitehorne-Smith, Robyn Martin, Daniel Oshi, Wendel Abel, Ben Milbourn, Kristen Smith, Sharyn Burns

**Affiliations:** 1 School of Public Health, Curtin University, Bentley, Western Australia, Australia; 2 School of Global, Urban, and Social Studies, RMIT University, Melbourne, Australia; 3 Department of Community Health and Psychiatry, University of the West Indies, Kingston, Jamaica; 4 School of Allied Health, Curtin University, Bentley, Western Australia, Australia; Faculty of Health Sciences - Universidade da Beira Interior, PORTUGAL

## Abstract

**Background:**

People with comorbid mental illness (MI) and chronic physical illness (CPI) face a range of health and quality of life challenges. The appropriate screening and management of comorbid MI and CPI are crucial to improving outcomes for this population. Despite this, there is a dearth of research exploring the health system response to the screening and management of patients with these comorbidities in public primary care settings, in several jurisdictions including Jamaica. This study explored and described the attitudes, perspectives, experiences, and practices of policymakers, primary care physicians, psychiatrists, and mental health nurses regarding screening and management of comorbid MI and CPI.

**Method:**

Twenty-nine participants representing policymakers, primary care physicians, psychiatrists, and mental health nurses took part in semi-structured interviews. Data was collected over the period April to November 2020 and subject to thematic analysis.

**Results:**

Three overarching themes emerged from the data related to: 1) Policies and Protocols; 2) Clinical Practice; and 3) Personnel. The interplay of these themes illustrated fragmentation and gaps between national policies and guidelines and clinical practice. The findings also identified factors related to personnel, including barriers that limit clinicians’ abilities to adequately screen and manage this patient population.

**Conclusion:**

There is a need for the continued development and revision of policies and protocols that support integrated care for patients with comorbid MI and CPI in primary care settings in Jamaica. Additionally, programs and strategies to improve clinicans knowledge, skills and access to resources are necessary to help them offer improved quality of care around screening and management for this patient population.

## Introduction

The bidirectional relationship between mental illness (MI) and chronic physical illness (CPI) is a longstanding concern [1, 2]. People diagnosed with severe and enduring mental illnesses (SEMI) such as schizophrenia, are more likely to develop CPIs such as diabetes, and cardiovascular diseases [3], indicating a 10–25 year reduction in life expectancy and a higher mortality rate than the general population [2, 4]. Similarly, people with CPI are prone to developing common MI such as depression and anxiety [5] resulting in increased levels of disability and poorer functioning, quality of life, and health outcomes [1].

Within the Americas, non-communicable diseases (NCDs) are the leading cause of morbidity and mortality, accounting for 80% of deaths [6]. In the Caribbean, an estimated 37.4% to 64% of MIs are undetected and untreated [7]. In Jamaica, the estimated prevalence of depression among the adult population is 14.3% [8] with the predicted economic burden of $2.7 billion (USD) between 2015–2030, representing a greater economic burden than any other NCD, except cardiovascular disease [8].

To address increasing NCDs, early detection and management through screening are vital [9] especially through the integration of mental health with primary care [10]. As the first point of contact, primary care is considered ideal for screening and management of physical and mental illnesses [4, 11]. In addition, due to the broad scope of illnesses seen in primary care, World Health Organisation [WHO] has reported there to be less stigma and/or discrimination associated with visiting primary care physicians (PCPs) [4, 11].

In keeping with recommendations outlined in the 2001 World Health Report, Jamaica fast tracked changes to policies and legislation to incorporate screening and management of NCDs including common MIs into primary health care [12, 13]. According to the current Jamaican National Strategic Plan for Non-Communicable Diseases, PCPs operating within the public service are expected as part of standard practice, to screen and detect symptoms of major depressive disorder and other common MIs, initiate management and refer to mental health services [14]. Jamaica purports an integrated and collaborative model of care, which involves cooperation and coordination of patient care across multiple health care providers who address their mental and physical health needs [13]. This is facilitated through mental health services being situated within 139-community health and inpatient sites, and aims to improve access to mental health services, reduce stigma and strengthen the management of comorbidities [13].

Despite these policy advances in Jamaica, there is little published research showing its translation into practice with one recent study suggesting that 66.7% of PCPs are either inadequately trained or lack the confidence to detect and manage MI such as depression [15]. Consequently, the present study sought to explore and describe the attitudes, perspectives, experiences, and practices of health policymakers and clinicians regarding the screening and management of patients with comorbid MI and CPI in primary care settings in Jamaica.

## Materials & methods

### Study design

This qualitative study is part of a larger mixed-methods study identifying the enablers and barriers experienced by people with SEMI when accessing health care for CPI in Jamaica [16]. The current study utilised a qualitative research design, with social constructivism as the theoretical framework, which allowed for in-depth exploration of the topic and consideration of the social processes and interplay involved in the research exercise and emerging findings [17].

### Setting

The study was conducted in Jamaica, the largest island in the English-speaking Caribbean, which is classified as an upper-middle income economy with an estimated population of over 2.96 million [18]. Jamaica’s public health system utilises a community-based primary health model delivered through 322 community health centres. Services provided in these centres vary and include curative and specialty clinics with the former operated by PCPs who address general health concerns [19]. Specialty clinics focus on areas such as dental, maternal, child, and mental health [20]. Mental health clinics operate at primary care facilities throughout the island and are managed by psychiatrists and mental health nurses [13]. A referral system exists between clinics operating at the same primary care facility [20]. Secondary and tertiary care are offered in 23 hospitals throughout Jamaica [21].

### Participants

The current study included health policymakers (HPMs), PCPs, psychiatrists (Psychs), and mental health nurses (MHNs) working in the public health system for at least one year, or in the case of Psych at least in their final year of residency. Those with less than 12 months experience in their role within the public health system were excluded. Purposive and snowball sampling techniques were used to recruit participants through emailed invitational flyers and sensitization sessions in professional networks and community health clinics. Individuals who expressed interest in participating in the study were followed by via email or telephone calls.

### Data collection

**Fig 1:**
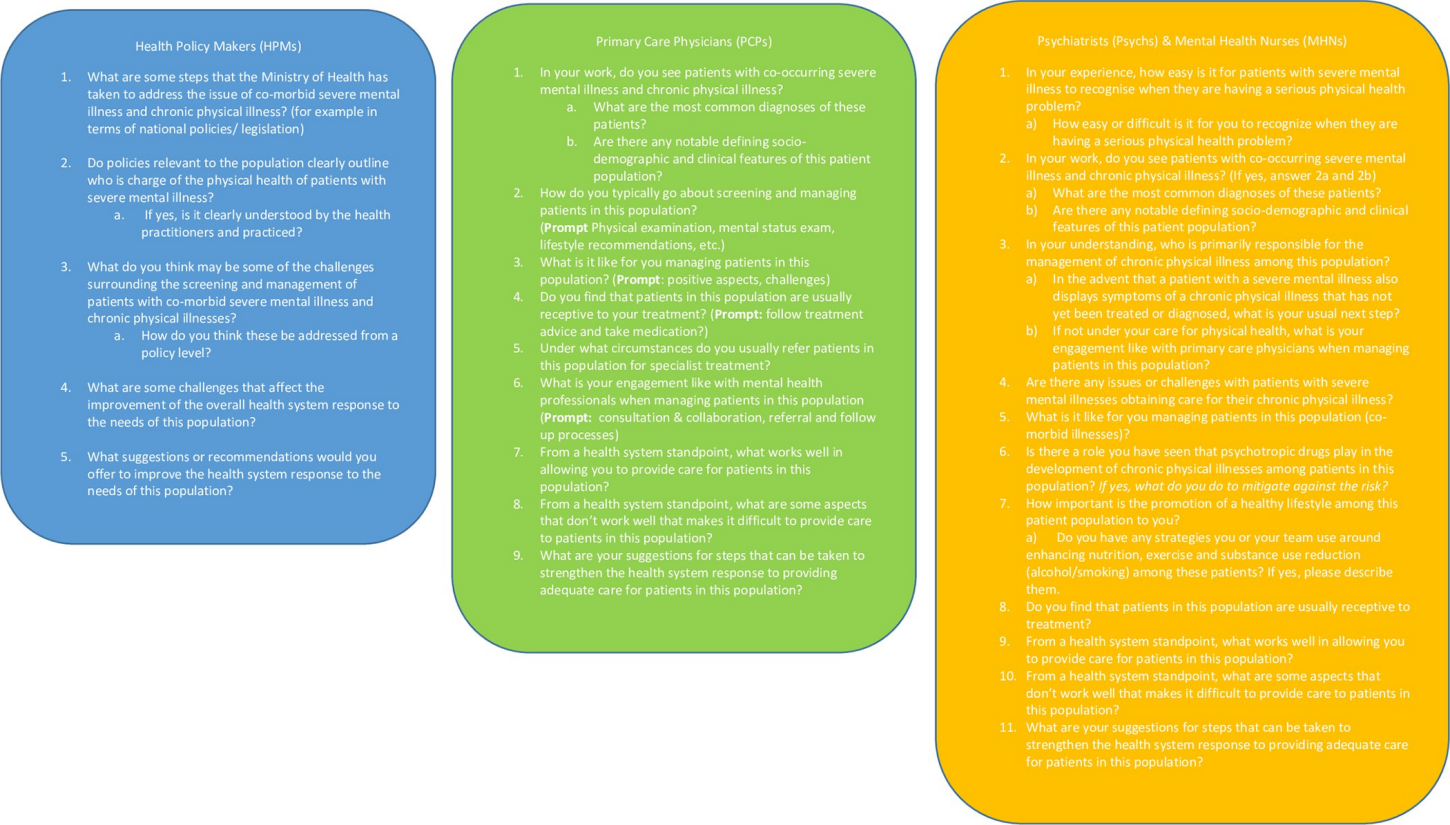
Interview questions for participant groups.

Data was captured over from March to November 2020, using semi-structured interviews, allowing a guided exploration of specific areas and dialogue on issues nominated by the participant [17]. Written informed consent was obtained and audio-recorded interviews ranged in length from 30 to 60 minutes. Interview guides exploring aspects of screening and management practice for the patient population were utilised to collect data (Fig 1).

Due to the impacts of the COVID-19 pandemic [22], only one interview was conducted with each participant. In addition, interviews were primarily conducted via video conferencing or telephone.

### Data analysis

All interviews were transcribed verbatim from audio recordings and returned to the participants for review. A thematic analysis was conducted [23] and N-Vivo v. 12 software used to manage and guide the analysis [24]. Multiple detailed reviews of all transcripts were conducted and initial codes generated from a line-by-line analysis of each transcript [23]. The emerging codes were reviewed by two authors, allowing for the identification of themes, which were further defined and named, all themes were derived from the data and the report produced [23]. To control for the variances in the interview guides, specific interview questions were used for comparison, covering knowledge of policy directives, the role of clinicians in the screening and management of comorbid MI and CPI and perceived challenges health professionals encountered in their practice. The number of interviews were deemed adequate and, data saturation achieved as codes became repetitive across later interviews [25]. Memos were written and reviewed throughout the analytic process to aid in the synthesis of emerging findings [23], with findings presented thematically based on the scope of the study.

### Rigour

The lead author who is a trained Jamaican Psychologist conducted all the study interviews and led the analysis process. She worked in mental health treatment research for over fifteen years prior to conducting this research. She was interested in this area as she observed barriers to people with severe and enduring mental illnesses receiving appropriate care for their physical health. She acknowledges that her prior experiences and assumptions could affect the research process. In addition, given the small niche of professionals working in this area in Jamaica, she was familiar with several of the study participants through prior working relationships. All participants were aware that this study was being conducted as a part of a doctoral thesis. Given these factors, several measures were implemented throughout the research process to ensure the trustworthiness of the study.

Firstly, the lead author engaged in bracketing of her own views and position [17]. The ability to bracket and critically assess the research process and findings was enhanced by engaging in reflexive discussions with the research team, which consisted of multi-disciplinary and multi-national team members [17]. This allowed space for the research team to identify and clarify areas of potential bias and engage in multiple reviews of all data points [26]. Direct quotations from participants were used throughout the study findings to ensure the credibility of the results. Data source triangulation across four participant groups also supported the credibility of study findings.

A reference group (n = 6) consisting of health research academics and representatives from the participant groups of the broader study provided oversight of the research process. This group provided face validity for the interview guides, as well as reviewed and provide feedback on the data collection strategy and the study results and conclusion. Their involvement assisted in reducing the risk of bias and enhancing credibility, dependability and confirmability of the study [26]. Additionally, the transferability of findings was ensured by capturing thick descriptions from participant groups who worked in various capacities across Jamaica.

### Ethics

The study received institutional ethical approval from the Curtin University Human Research Ethics Committee (HRE 2020–0022), the Ministry of Health and Wellness Jamaica’s Medico-legal Ethics Committee (2019/49), and the University of the West Indies, Faculty of Medical Sciences Ethics Committee (#ECP 101, 19/20). All participants provided verbal and written consent.

## Results

Overall, 29 participants comprising four HPMs, nine PCPs, eleven Psychs, and five MHN contributed to the study. Twenty-one participants identified as female and eight as male, all participants were 25 years or older. Most had been working in their current role for 1–5 years (n = 11), others for 6–10 years (n = 10), and the remainder for over 10 years (n = 8).

### Themes

**Fig 2:**
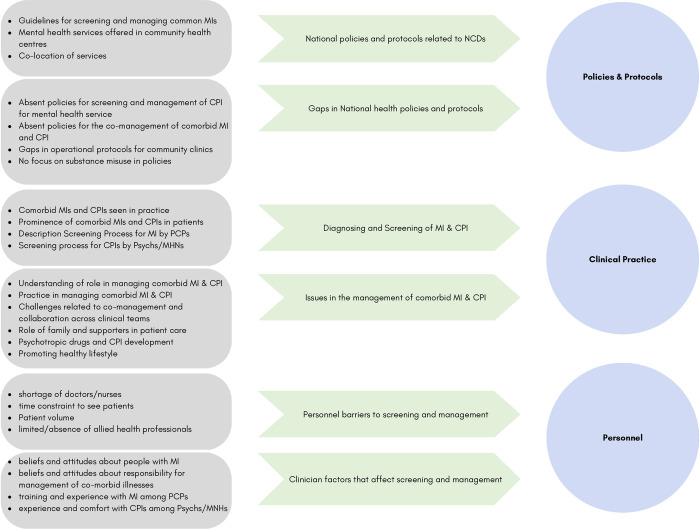
Illustration of summarised coding tree- examining the diagram from left to right shows the coding process of how identified codes were named, organised and grouped into thematic areas.

The data analysis process resulted in the identification of several levels of codes that were organised and categorised into three overarching interrelated themes that shape the screening and management process for comorbid MI and CPI in primary care settings in Jamaica: 1) Policies and protocols; 2) Clinical Practice; and 3)Personnel. Fig 2 represents a summarised version of the final three levels of the coding process, which involved grouping similar or related codes to generate the three main themes.

#### 1) Policies and protocols

The theme Policies and Protocols considers national policies and protocols related to screening and management practices. Participants indicated that the focus of the national health policies and protocols for managing comorbid MI and CPI emphasized the role of PCPs. Health policymakers (HPMs) referred to the current Jamaican National Strategic Plan for Non-Communicable Diseases [14] that identifies that PCPs are responsible to screen for MI in people with CPI:

*“A part of that process (primary health care) is to ensure that persons with chronic illnesses*, *they should be screened for mental illnesses*… *and once persons are identified…the protocol is for them to be referred to the mental health team to ensure that they’re getting that kind of specialized care* ***…****So the primary care physician does not generally manage severe mental illnesses*. *So they are trained to manage depression and mild (mental) conditions and if they are unable to manage the cases adequately then persons are referred to the mental health team for further care*.*”* **(HPM #1)**

The policies and protocols outlined for PCP specify them as the first point of contact and mental health practitioners (psychiatrists and mental health nurses) as secondary contacts:

*“Actually what I would say that it’s more primary care physician who would have to be the one who’s on the front line to pick up either physical or mental issues because they are the ones who see more regularly and they are the first point of contact into the primary care system*. *Yes*, *the mental health practitioner would have to make sure in their clinic when they are seeing patients for mental illnesses they remember that there may also be physical disorders that may develop with time that they need to look out for but I think the primary care physicians are the ones who will naturally see most of the patients and be the first point of contact…”* **(HPM #2)**.

Although there was an acknowledgment of mental health practitioners (Psychs and MHNs) needing to recognise patients’ physical illness, participants were unaware of any clear policy directive related to their role in screening and management of CPIs. This was noted as an area of a significant policy gap as it represents a loophole for patients who do not access PCPs as their first point of contact. Consequently, the core of the policy issues expressed by participants was the need for a comprehensive and inclusive assessment policy that involves all relevant health practitioners in the screening and management of comorbid mental and chronic physical illnesses:

*“My stance is that there should be a policy that looks at both physical health and the mental health of the person as a part of the comprehensive assessment… the comprehensive approach is what is needed for all patients”*. **(HPM #2)**

Despite the recognised gap in national directives for mental health practitioners in the management of CPIs for their patients with MI, a few Psychs referenced international guidelines that guide their practice. These guidelines were developed to help mitigate the increased risk people with SEMI face of developing CPIs due to being treated with psychotropic drugs:

*“Well initially according to international guidelines you should be taking a detailed history to again check if they are predisposed or have a strong family history or if they themselves have a personal history by just being obese or having a sedentary lifestyle*. *So we really should be exploring the benefits vs the risks at the beginning before prescribing medication and then if it is that this is the absolute last resort*, *then we’re supposed to be monitoring them so that we have routine investigations that will monitor their blood pressure*, *weight*, *height*, *waist circumference among others*. *So there are guidelines…”* **(Psych #1)**

Another area of concern noted by several participants was the absence of national policy guidelines and protocols on the routine screening and management of substance use problems from both the Jamaican National Strategic Plan for Non-Communicable Diseases and the drafted National Mental Health strategic plan. PCPs and mental health practitioners expressed a lack of detailed screening or active dialogue about substance use with their patients. They also expressed a lack of knowledge about resources available for assisting clients with substance use issues:

*“I don’t think primary care doctors are trained enough to know*, *how*, *when*, *and who to refer them (patients) to for substance abuse disorders…if you stop any clinician and ask them what do you do with somebody who has a substance abuse problem they’re going to look up in the sky and scratch their head*, *I promise you*.*”* **(PCP #5)**

As it relates to the management of patients with comorbid MI and CPI, participants explained that the organisation of the community primary healthcare system involves the co-location of curative and mental health clinics. This aspect of the health system response is advantageous in helping streamline services and improve patient access to care. However, there was an absence of national-level guidelines related to how clinic administrators can strategise offering services in ways that best meet the needs of patients. For example, in some settings, mental health clinics are held on different days from the curative clinic. This presented a gap in the operational protocols:

*“One of the good things though*, *the mental health service is also offered in primary care so it’s not necessarily a referral into the secondary care system*. *So even though the person is referred to another service*, *that person is still given an appointment within the chronic care clinic or the mental health clinic for follow-up and I suppose that’s where the gap is*… *How do we ensure that we have both services available on the same day so that persons just take one day and get holistic care for those conditions*?*”* **(HPM # 1)**

This was of particular concern as HPMs discussed issues related to social determinants of health and recognised that people with comorbid MIs and CPIs who access the public health service often lack financial and social resources to attend clinics on multiple days. This lack of coordination between clinics was seen as diminishing some of the advantages of co-location and creating additional barriers to accessing care.

Furthermore, this created a barrier to what participants described as co-management as PCPs and mental health practitioners may not meet due to different clinic dates. Health policymakers suggested co-management is an important aspect of the collaborative care model. Nevertheless, clinicians and HPM were unaware of specific national guidelines or protocols related to how co-management should occur. PCPs were clear that they had a responsibility to manage CPIs, screen for MIs, and refer patients to mental health services. Yet, their role in follow-up and engagement after referral to the mental health team was not articulated. Similarly, mental health practitioners expressed varying views about their responsibility in the management of CPIs, referral, and follow-up.

#### 2) Clinical practices

The theme of Clinical Practices involved discussion around participants’ perception of the prevalence of comorbid MI and CPIs, and the application of prescribed policies and protocols in screening for and managing these illnesses. Participants engaged in clinical practice were asked about their perception of the prevalence of co-occurring MI and CPI. Primary Care Physicians estimated that between 5–25% of their patients had comorbid MI and CPI, commonly involving Schizophrenia, Major Depressive Disorder, Bipolar Disorder with, Hypertension, and/or Diabetes Mellitus. Mental health practitioners estimated that between 10–60% of their patients had comorbid MI and CPI, involving Schizophrenia, Schizoaffective Disorder, Bipolar Disorder accompanied by Hypertension and/or Diabetes Mellitus.

**Fig 3:**
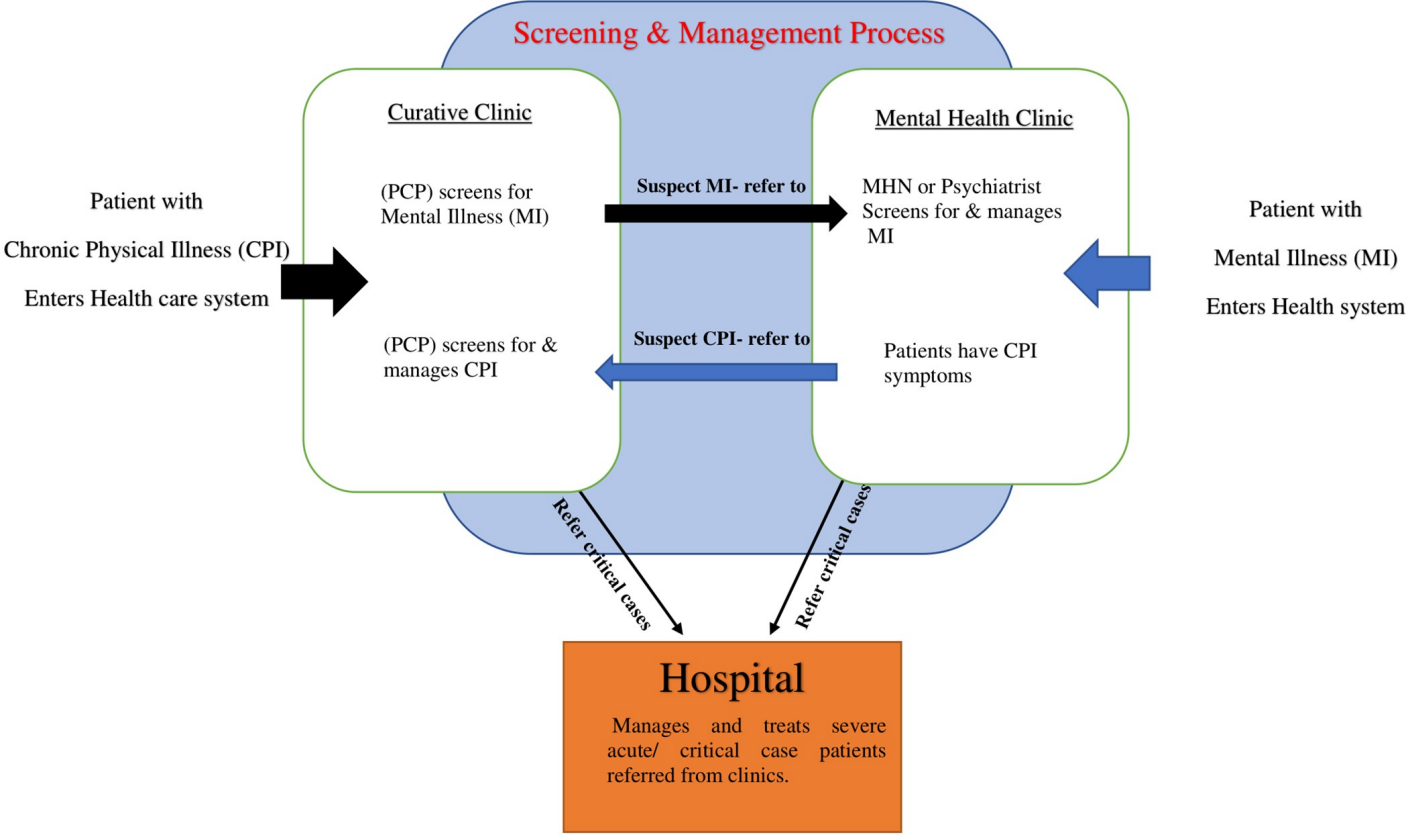
Screening and management process for patients with MIs and CPIs in primary care.

Primary care physicians and mental health practitioners outlined their experience of the screening process in clinical practice as shown in Fig 3.

Primary care physicians reported a lack of consistency in screening approaches for MIs across clinicians and clinic sites. Most PCPs reported that depression was the only mental illness screened for in a standard way. At some health centres, there were clear protocols for depression screening:

*“At one health centre… they developed mandatory questions for us to ask those patients (with CPI) to screen them for depression… the nurse would stamp the two criteria questions for depression (Patient Health Questionnaire-2) in the patient’s docket before the doctor would see the patient and then they will be screened for depression before the doctor would continue with their visit with the patient*.*”* **(PCP #3)**

While in other settings, depression screening was done at the discretion of the PCP’s clinical suspicion of the presence of a MI in a specific patient.

“We will go through their history on what the presenting complaint is… particularly patients who you might now be suspecting (of mental illness)” **(PCP #6)**

Screening tools were reportedly used by most PCPs to assist in their assessment of common MIs. These tools included the Patient Health Questionnaire two or nine [27, 28], the General Anxiety Disorder Assessment [29] and the Mental Status Examination or mini-mental status examination. One PCP also reported using the Hamilton Depression Rating Scale [30]. Other mental illnesses were identified based on clinical observation of patient presentation and history taking:

*“We would have that patient assessed in terms of a risk evaluation and then would have that patient actually worked up to rule out other medical causes*, *you know of what symptoms the patients are presenting with… then having done that and finding no medical cause that would explain the symptoms we would then would refer to Psychiatry clinic*, *but if it’s a case that the patient has been evaluated as having somewhat elements of suicidal or homicidal thoughts*, *or intentions*, *then (we) would refer that patient to a critical-care/accident and emergency unit*.*”* **(PCP #5)**

It is noteworthy that PCPs reported that most of their patients with MIs were previously diagnosed and treated by the mental health service, and referred to the curative clinic for screening and management of physical health problems.

Additionally, screening for alcohol and other drug use was not a standard part of the practice for most PCPs. Less than half of PCPs reported any screening practice and for those who did report screening, it was at the point of the initial interview for history taking but was not routinely monitored or treated:

*“If it’s a new patient that we don’t know anything about then we would ask about*, *you know*, *alcohol or drug use*, *but otherwise*, *there wasn’t any screening*, *you know for the regular follow-up patients we didn’t really screen that often for substance use*.*”* (**PCP #3**)

As it relates to screening practices for CPIs among mental health practitioners, most did not routinely screen for CPIs but indicated they did screen if they felt it was warranted. Clinical observation and patient reports of symptoms were used to guide when further investigations into physical health concerns were needed:

*“…you should not dismiss what the person will say just because he had a mental illness*. *So if due diligence is done and you see that okay*, *this is actually possible*, *then it kind of prompts you to investigate further”*. (**Psych #5**)

Mental health practitioners who engaged in screening for CPIs explained that:

*“We usually would fully assess*, *do a physical examination*, *take a full history and the next step from there would be to make a referral as I said to be Internal Medicine service*. *We tend to take off the blood works that we need to do*, *prior to making the referral*, *just so that it facilitates the speed of them being seen in the other service*.*”* **(Psych #9)**

However, a few psychiatrists expressed the view that screening for CPIs did not fall within their purview, for them there was a clear delineation of their role to treat MIs and not CPIs:

*“We’re not really checking them for (blood) pressure and so if they come for mental illness*, *that’s it*. *They go to another doctor for their pressure (blood pressure) and sugar (glucose levels)… so we refer them to the appropriate physician or appropriate doctor to deal with that”*. (**Psych #10**)

Additionally, mental health practitioners indicated that the screening process for CPIs was complex because it was dependent on the patient’s ability to articulate physical health concerns. There were varying viewpoints about the ability of patients with serious mental illnesses to report on physical health problems. While a few mental health practitioners reported that patients with severe and enduring mental illnesses are capable of reporting on physical health concerns, the majority believed patients might not be able to explain their symptoms:

“*It’s not easy for them (people with SEMI)…some of them are kind of out of touch with their whole body system and so on and it really not*, *I would say a majority of them don’t know*. *A few will know but for the majority*, *it is not so easy to recognize*.*”* (**MNH #4**)

In cases where patients had difficulty articulating their symptoms, mental health practitioners emphasised the important role family or supporters play in sharing their observations and advocating for the patient:

*“A lot of times you’d find that when a patient has a severe mental illness*, *it’s the relatives that are the ones that are pushing for their treatment in the mental health aspect and they may be the ones that are pushing for the treatment on the physical health aspect as well*. *So it really depends on whether or not they have a good support system as it relates to that*.*”* **(Psych #2)**

Despite this, mental health practitioners reported that some physical health problems that were not readily observable through an external examination could be missed due to poor access to resources such as equipment (e.g. scales or blood pressure machines), adequate support staff, and patient load on clinic days at some clinic sites. In addition, COVID-19 has further complicated the screening for CPIs among this population, for example, some routine checks were temporarily discontinued to limit the use of the equipment and the potential spread of infection:

*“For you to be sanitized and so*, *you know your blood pressure machine on everybody*, *after two or three persons*, *it is going to be soaked*. *So we took the decision of not doing this*. *However*, *it is available for persons who we suspect might have need*, *once we see any physical issues…it is there that we can pop out and say we’re going to do that (blood pressure check)”*. (**MNH #3**)

Mental health practitioners emphasised that to mitigate the potential of missing CPIs in the mental health clinics, they encourage patients to do a complete physical health check-up annually with their PCPs.

Another consideration affecting adequate screening and management practices for patients related to communication between clinical teams. Although the services of the curative clinic and mental health clinic were co-located, PCPs and mental health practitioners expressed mixed views related to ease of communication within the public health primary care system. In some clinics, co-location provided better access to medical records held at clinics within the same facility and referral of patients. A few PCPs reported good engagement and communication across curative and mental health teams. However, most participants reported that engagement of clinicians across clinics was limited or non-existent and the system relied heavily on patients to carry messages from physicians:

*“Not much of a process in terms of the public system*. *Most of the time it’s the patient that comes and says ‘I went to that other doctor and this is what they gave me or this is the prescription’*… *in the public system*, *you don’t usually get any correspondence from the colleague*, *it’s the patient that’s the means of communication*. *They’ll come back and say well that doctor told me X or Y*. *The process itself needs some improvement*.*”* (**Psych #9**)

Likewise, some PCPs described their engagement with the mental health service as generally uncoordinated, with poor collaboration, little communication, and no follow-up after patients were referred:

*“It’s basically like two people managing separately*, *we don’t sit and talk and say*, *‘let’s talk about Anna Jones*, *how is Anna Jones doing*?*’*…*not much of a collaboration around managing patients and I understand because there are a lot of patients and for the most part*, *although it is a team effort*, *we do our own thing- I take care of the (blood) pressure- you take care of the schizophrenia- it is not we take care of her together”*. **(PCP #4)**

Although communication was potentially enabled through co-located patient records, there was no system to share medical records outside of the co-located services, including with hospitals or other health services. There was also a noted absence of a computerised system or database to access and store patient medical records. This resulted in patients being asked to courier letters and information between clinicians and/or being requested to present with their written prescriptions or medications. Some clinicians and health policymakers reported this method to be ineffective, as patients often provided incomplete information and forgot the names of their prescriptions or medications. It was recommended that these processes be addressed in both policy and practice:

*“We need to… really reorient and re-educate our practitioners to understand the whole concept of NCDs and mental disorders being closely intertwined and that they need to address both aspects of the patient is very critical for the overall outcome… we don’t (need to) have separate silos where the doctors that deal with mental illness are over that side and the doctors that deal with curative illnesses are over that side… but there is collaboration through maybe case conferences or consultation to deal with the patient so that both sides can get comfortable dealing with physical illnesses and dealing with mental illnesses*.*”* **(HPM #2)**

That observation was elucidated by the finding that most PCPs did not consider they had a significant responsibility to manage MIs outside of mild to moderate depression. Even then, some PCPs shared their observation that they had encountered PCPs who did not do this and automatically referred symptomatic patients to the mental health clinic without any treatment intervention:

*“They (some PCPs) refer everybody…if you say you’re not sleeping*, *they didn’t want to ask you why you’re not sleeping-sometimes they refer them (patients) without even doing blood work investigations*. *This is how quick they refer them*.*”* (**PCP # 5**)

However, for those PCPs who engaged in the management of MIs, this mainly took the form of prescribing antidepressant medication and providing basic counselling. Medication management involved prescribing anti-depressants and paying close attention to possible medication interactions for co-morbid CPIs. Primary care physicians discussed that patients who reported not feeling any notable improvement in symptoms by the second visit were referred to the mental health clinic. Patients deemed to need urgent medical intervention were referred for critical care through the accident and emergency department at general hospitals.

Among mental health practitioners, there was some discrepancy in the views of psychiatrists and mental health nurses as it related to their role in managing CPIs in their patients. Most psychiatrists stated that the management of CPIs was the responsibility of the PCPs, despite their acknowledgment of the role that psychotropic drugs play in the development of CPIs among patients with severe and enduring mental illnesses:

*“…some of the newer medications do predispose them (patients) to get diabetes or hypertension*, *which are two of the common ones that we really have and obesity as well as the fact that their mental illness could also lead them to have poor physical health as well*. *So it’s a combination of things it’s hard to say which one but whatever it is*, *there is a correlation between the two in terms of the physical illnesses and a mental illness*.*”* (**Psych #1**)

For these psychiatrists, management of CPIs involved mainly screening and then referring; some would initiate management depending on the nature of the illness and then refer to the relevant clinic for follow-up. In contrast, mental health nurses mostly believed the management of CPIs was their responsibility based on the level of engagement and follow up they have with the patients:

*“Well*, *it’s the nurses (responsibility to manage CPIs)*, *because the doctor will diagnose them and give them their prescription and you have the nurse to do the education… I have to try to ensure that I do my follow-up and get them to keep to the appointment… the nurses are there from 8 am giving the patient’s a promotional talk every time… you do their blood pressure*, *their weight check*. *You counsel them*. *Because even after the doctor has spoken to them*, *they come right back to you*, *the nurse*, *for clarification*. *You also take out the medication and show them and give them explanations…*” (**MHN #1**)

All mental health practitioners and some PCPs supported the promotion of a healthy lifestyle as a critical to the management of patients with these comorbidities as well as to mitigate the physical health risks associated with psychotropic drugs. Healthy lifestyle promotion was done through individual, family, and group health talks, referrals to dietitians or nutritionists, and focused on raising awareness about diseases, self-care tips, and encouraging appropriate diet, rest and physical activity.

#### 3) Personnel

The theme of Personnel outlines findings related to clinician characteristics or staffing issues that affect screening and management practices. A number of personnel challenges were noted across participant groups that were believed to affect clinicians’ ability to conduct routine comprehensive screening of patients with comorbid MI and CPI.

Clinicians expressed that insufficient staff in curative and mental health clinics negatively affected the management of comorbid MI and CPI in patients. The quality of care offered to patients is often compromised as the time allotment for each patient is reduced to enable staff to see all attending patients:

“*As I say the shortage of staff is my problem*. *Sometimes when I could spend a little more time talking with a patient*, *as talk therapy helps more than the medication*. *But that had to be reduced to 3–5 minutes now*, *as I have over 35 patients in one day now*, *and I am alone*. *Also*, *if they wait too long they (patients) will leave this clinic and go away*. *You can’t afford that because when they go away*, *sometimes you don’t find them*.” (**MHN #2**)

Staff shortage coupled with large patient volume greatly hindered mental health practitioners’ ability to adhere to protocols related to mitigating risks associated with psychotropic drugs:

“M*ost of the time they’re not adhered to (guidelines for mitigating risk of psychotropic drugs) and that’s the honest truth because in the clinic setting in the public system*, *we are limited*, *in that we don’t have the nursing staff at times to assist with taking the measurements that we may need*. *Again*, *the clinic numbers may be large*. *Patients are outside getting frustrated*, *so you want to try to move them through as quickly as you can*. *So there are limitations*. *So oftentimes no*, *the guidelines are not adhered to as strictly as they should be”*. (**Psych #9**)

In some settings, Psychs and PCPs were absent and MHNs and psychiatric nursing aides routinely oversaw the clinic activities including performing the necessary vital checks and encouraging patients to follow up with their PCP for any CPIs:

“*There are a few clinics now that we don’t have any doctor in as in working in Psychiatry…(in these cases) we encourage them (patients) to maintain their follow because we’re aware that they were already following up*, *sometimes in the same Health Center where we are”*. **(MHN # 1)**

The issue of staff shortage and large patient volume were also of notable concern to PCPs. They shared that the sheer volume of patients that access clinic services rendered it very difficult to engage in a collaborative care approach. High patient volume instead resulted in reduced time with patients, limited available time for clinicians to collaborate, discuss and follow up on individual patients:

*“Clinics are too busy and too packed*, *because for you to pick up the phone and call whoever it is- and remember (mental health) clinic is only one day for the week- because the mental health team has a lot of all that stuff doing*, *they have home visits*, *they have emergency calls*, *they have clinic at the hospital*. *They have clinics on different days and at different facilities*.*”* (**PCP #5**)

Additionally, some participants linked the strain experienced by clinicians to the heavy focus of the health system response to the role of medical staff such as doctors and nurses. Only a few participants noted the presence, albeit limited, of psychologists and social workers. However, these professionals were not referred to in the standard process of screening or management of patients with comorbid MI and CPI. Several participants spoke to the need for multidisciplinary allied health teams that can help clinicians to better manage patients with comorbid MIs and CPIs as these professions could provide talk therapy and social interventions for patients.

Some clinicians felt that allied health professionals could help alleviate some of the pressure placed on the medical staff as well as help to address other social determinants of health that may be negatively affecting patients and contribute more than just medication management:

*“Some of them (patients) need occupational therapies*, *social workers*, *and psychologists*. *(These professionals) are needed to help to mobilise these persons manpower and resources are lacking and if we had them we would see a vast improvement in the welfare of these clients and so we could see a support system that could help them have a better life…”* (**MNH #5**)

Furthermore, the role of clinicians’ previous training, direct experience and attitude towards screening and management for MI and/or CPIs was noted to be important to their clinical practice. Among PCPs, those who had undertaken additional training or who had experience working with people with MI argued that providing medical care for people with comorbid MI and CPI was the ‘same’ as any other group. These PCPs also indicated they enjoyed the work:

*“I would say that it was fine for me (working in the clinic with patients with MI) because I had some background*. *I had worked 4 months as a SHO (Senior House Officer) assigned to mental health*, *so I had some background (in mental health)*, *so it wasn’t really very challenging for me*.*”* **(PCP #3)**

Further, this small group of PCPs expressed care by spending ‘more time’ with this population, listening to their concerns, offering support, educating patients, initiating treatment, and taking time to explain treatment and referrals in plain language:

*“(PCPs) are you willing to take the time to educate them (patients)*?…*if you give them the chance and empower them…(talk to them) in a language that they can understand and you work with them and make them feel safe then they will come back and you don’t have to teach them that much”*. **(PCP #7)**

These PCPs also referenced ‘holistic care’ and the need for a multidisciplinary approach, which they believed, should involve psychologists and social workers. They described good working relationships, which included regular dialogue and collaboration with Psychs via telephone or in-person. All participants who reported a good relationship with the mental health team had additional training or experience in mental health:

*“If I am suspicious of this patient being depressed or having schizophrenia*, *then I consult the psychiatrist*. *I always*, *always involve my psychiatrist because first of all*, *that I do have some experience in the area but I am not the specialist…so it’s always a collaborative effort for me and you know collaborative team approach*.*”* **(PCP #8)**

This difference was even observable to some mental health practitioners. They noted that patient-centered PCPs tended to be more motivated to engage them:

*“(Engagement) is still dependent on the motivation of the particular physician*. *So depending on the particular setting that I’m in*, *some physicians are just very thorough and they’re very patient-centred*. *So they will initiate contact with me as much as I will initiate contact with them*, *but then there are other settings where they just don’t have that same drive or motivation*. *So you will not have that sort of engagement*.*”* (**Psych #4**).

In contrast, PCPs without additional training and experience tended to describe the population as ‘challenging’ and said they were ‘uncomfortable’ treating this group of patients. Primary care physicians in this group framed this in terms of limited professional confidence, exacerbated by a sense that psychotic patients are irrational and unstable. Consequently, this group of PCPs argued that the management of patients with MI was not their responsibility. They also reported that they immediately referred people who demonstrated psychiatric symptoms to the mental health service without detailed investigation or intervention. They reported a sole focus on the CPI aspect of care for patients with MI:

*“If it’s severe depression*, *no we don’t deal with that… we just see them for their chronic disease*, *and we don’t address the mental illness…some (PCPs) are not comfortable with it… I am fine with depression*, *to be honest*, *the ones who are diagnosed with schizophrenia or psychotic disorder*, *I am a little hesitant*, *I will see them but when they are unstable I am hesitant*…*they can be unpredictable…they can be irrational*. *If they are in front of me and stable I am fine but if I pick up on any sign of instability I am uncomfortable*.*”* **(PCP #4)**

Among mental health practitioners there were mixed views and practices. Mental health nurses expressed overall comfort managing the CPIs of patients as guided by practitioners, however, and this was different for psychiatrists. Psychiatrists who reported extensive experience in general medical practice or who expressed comfort and confidence in their ability to treat physical illnesses favoured holistic care:

*“…it (treating comorbid patients) keeps my clinical skills up to scratch*, *current*. *It allows me to not just be limited to just Psychiatry*. *It gives me that opportunity to treat as a whole*. *And for me to utilize all other aspects of my training in dealing with these patients*. *It just allows me to be able to use my medical knowledge on a whole and treat them as well*.*”* (**Psych #6**)

These psychiatrists tended to report thorough physical examinations, investigation of physical health complaints, initiating management of CPIs, follow-up with PCPs and patients about CPIs, more careful mitigation measures against the risks of psychotropic drugs, and involving the family or support persons in care and collaboration:

*“I tend to make my assessment as psychiatric management …and I then tend to communicate that information to his primary physician or the person who made the referral*, *simply because*, *this allows the primary care physician to be aware of what (the) working diagnosis is*, *what medications you think are necessary*, *but also gives the primary physician a second opinion of the chronic medical issue that they may have been treating in the first place and help to possibly streamline (care)*… *So if you’re able to streamline that (care) what you find is you give the patient a better chance of dealing with both types of ailments*.*”* (**Psych #5**)

In contrast, Psychs who lacked current knowledge on the treatment of physical illnesses reported focusing on MI rather than physical concerns. They saw their role as one of encouraging the patient to follow up with their PCPs for CPI care and to take the medication prescribed to them:

*“Oftentimes the concept that if you’re a physician you should be able to manage everything*, *but some things go beyond the expertise of Specialists*. *So I wouldn’t bring it upon myself to manage somebody’s asthma and somebody’s diabetes because I didn’t think that that would be a disservice to the patient because that’s not what I have expertise in and other Physicians have expertise*.*”* (**Psych #2**)

## Discussion

Screening and management of comorbid MI and CPI are of global concern. In this study, there was a general acknowledgment across participant groups of the association between MIs and CPIs, with all clinicians reporting having patients with these comorbidities. Primary care physicians and mental health practitioners anecdotally reported that Diabetes Mellitus and Hypertension were the most common CPIs to co-occur with MI, an association that is commonly reported in the international literature [10, 31].

Across clinician groups, schizophrenia was anecdotally reported as the most common MI encountered in clinical practice. This finding represents a departure from international literature, which suggests depression to be the most common mental illness presented in primary care [4, 11]. This finding indicates the need for further quantitative studies that explore the prevalence of comorbid MI and CPI within primary care settings across Jamaica. Moreover, this finding is crucial as Jamaica national policies focus on depression as the primary MI encountered in primary care. Further research may suggest the need for a reorientation of policy and clinical practice around intervention strategies geared more specifically at patients with schizophrenia and CPIs.

Additionally, it is notable that HPMs indicated national health policies were crafted from the notion that PCPs are the first point of contact for patients with comorbid MI and CPI. However, all PCPs in this study identified mental health services as the most common first point of contact for these patients. This represents a significant area of disconnect between national policies and clinical presentation and practice. National policies and guidelines emphasise the role of the PCPs in screening for MIs but lack directives around their role in co-management of comorbid MI and CPI [14]. The policy guidelines also lack directives on the role of mental health practitioners in the co-management of patients with MIs and CPIs [14].

The finding that mental health services were the first contact point for people with co-morbid MI and CPI has significant implications for the role of Psychs and MHNs in management of CPI in this population. Especially since there were, mixed views among Psychs related to their responsibility in managing CPIs, with most indicating that screening for CPIs was not routinely performed. This is despite all mental health practitioners noting the link between psychotropic medication and the development of metabolic diseases. There have been recent calls for a shift in psychiatric practice for psychiatrists to expand their role in screening, managing, and promoting the physical health of patients with CPIs [32]. National consideration of this call and the inclusion of policies and guidelines to address this current gap in care is required.

Moreover, gaps in national health policy directives in these areas influence the continuation of a system that provides parallel channels of care for patients with these comorbidities [33]. Although the services of the curative clinics and mental health clinics are co-located they are not integrated. Co-location offers the advantage of shared access to medical records and in some instances improved access to services for patients [33]. However, without deliberate coordination between clinics, a collaborative care approach is unlikely to be fostered [34]. This was evident as most clinicians reported the services as being ‘separate’ with limited interaction between physical and mental health teams and poor or non-existent follow-up practices. This results in clear gaps in clinical practice as it relates to patient care [34].

Additionally, clinicians who were uncertainty about their responsibilities related to screening and management of MIs & CPIs demonstrated an overreliance on their personal beliefs, preferences, and experiences. Some PCPs and Psychs expressed lacking confidence and discomfort in treating patients in this population and tended to immediately refer instead of engaging in screening or management. This finding is consistent with other studies that have found that PCPs who lack confidence in treating patients’ were resistant to conducting detailed screening and management of patients with suspected MI [35, 36].

It is noteworthy that PCPs with post-study training and/or experience in mental health expressed a greater willingness to manage patients with MIs. The PCPs who were confident and comfortable with MI were more likely to undertake screening procedures and initiate management for MI. This group of PCPs expressed an attitude of care and concern for patients and tended to spend more time with them. They also reported more open communication and collaboration with the mental health team compared to other PCPs. This highlights the need for greater training and exposure opportunities for PCPs to work in mental health to develop the required confidence and competence [11, 36, 37].

Similarly, Psychs with prior experience in general practice and/or who believed managing physical health enhanced their practice were more likely to regularly screen for and manage CPIs than those who did not have this experience or belief. Consequently, there is a need for greater emphasis on improvements in primary care training for psychiatrists, especially around the screening and management of CPIs commonly found among patients with MI [32]. In contrast, all mental health nurses expressed a strong sense of responsibility for the overall health and well-being of patients, this may be due to the holistic way nursing is taught and practiced [38].

Several participants described personnel issues that limited clinicians’ ability to provide adequate management for patients with comorbid MI and CPI. These include limited staffing, high patient volume which reduced the time clinicians could realistically spend with patients as well as limited meaningful collaboration with colleagues. These barriers are commonly noted in health care systems in low-middle income countries with limited national healthcare budgets, and cultural and political will to address MI [39]. Additionally, the lack of emphasis of the health system response on a multi-disciplinary team approach inclusive of allied health professionals overburdens medical staff and reduces the quality of care offered to patients [40]. Allied health professionals are vital to assist with the mitigation of social determinants of health and improving patient outcomes [40].

### Strengths and limitations

This study involved gathering the perspectives of different groups of health care professionals allowing for triangulation of data. Participants were selected from across the island, which allowed for a broad range of perspectives to be considered. However, participants in this study were employed within the public health system, and therefore the findings of this study cannot be generalised to those working in private health care. Additionally, data were primarily collected via video and telephone calls, which may have resulted in some loss of data richness due to the reduction in observable non-verbal cues in the interview process.

### Conclusion

Jamaica has made progress in the incorporation of mental health care into primary health services. However, there are notable gaps and discrepancies between national policy guidelines and clinical practice for the screening and management of patients with comorbid MI and CPI within public primary care settings. The findings suggests the need for reorientation of health policies and guidelines to better align with what is observed in clinical practice for patients with comorbid MI and CPI. In particular, the finding that mental health services are the most common first point of contact for patients with comorbid MI and CPI. There is also a need for continued training and engagement of clinicians around the collaborative care model as well as investment in infrastructure, staffing and other resources to help improve the current health system response. Further quantitative research is needed to determine the prevalence and types of co-morbid MI and CPI in primary care to better meet the needs of this patient population in Jamaica.

## Supporting information

S1 File Inclusivity in global research. (DOCX)
